# *Garcinia cambogia* water extract alleviates insulin resistance and hepatic lipid accumulation in mice fed a high-fat diet

**DOI:** 10.29219/fnr.v67.8977

**Published:** 2023-03-14

**Authors:** Jinya Dong, Wen Li, Xiaocui Du, Xiaofang He, Bin Deng, Hongmei Zheng, Yang Tian, Jun Sheng, Chongye Fang

**Affiliations:** 1 Yunnan Research Center for Advanced Tea Processing, Yunnan Agricultural University, Kunming, China; 2 College of Agronomy and Biotechnology, Yunnan Agricultural University, Kunming, China; 3 Yunnan Rural Science and Technology Service Center, Kunming, Yunnan, China; 4 College of Food Science and Technology, Yunnan Agricultural University, Kunming, China

**Keywords:** Garcinia cambogia, insulin resistance, non-alcoholic fatty liver disease, obesity, hepatic lipid accumulation, hydroxycitric acid

## Abstract

**Background:**

Garcinia cambogia is widely used as a weight-loss supplement, and it is reported to be negatively associated with metabolic diseases including insulin resistance (IR), type 2 diabetes mellitus (T2DM), non-alcoholic fatty liver disease (NAFLD), and dyslipidemia.

**Objective:**

This study aimed to investigate the effect of G. cambogia water extract (GE) on high-fat diet (HFD)-induced obesity, IR, and hepatic lipid accumulation.

**Design:**

C57BL/6 male mice were fed HFD with or without GE, GED and GEP for 16 weeks, and the mice were subjected to insulin tolerance tests and liver histological analysis. The hydroxycitric acid (HCA) levels of GE, GED, and GEP were measured by high-performance liquid chromatography.

**Results:**

The results showed that GE significantly reduced HFD-induced body weight gain (*P* < 0.001), alleviated IR (*P* < 0.01), reduced serum total cholesterol (TC) (*P* < 0.001), and attenuated HFD-induced hepatic lipid accumulation. To investigate the constituent that was responsible for these effects, we separated GE into the component that dissolved in ethanol (GED) and the component that was precipitated by ethanol (GEP). Further mouse experiments showed that both GED and GEP were effective, but GED (which was used at a dose of 4 g/L) was more effective than GEP (which was used at a lower dose of 1 g/L). The HCA levels in GED and GEP were similar, although less than in GE. HCA may be the effective component in GE.

**Conclusion:**

This study provides evidence that G. cambogia can be used as a natural supplement to alleviate IR and hepatic lipid accumulation.

## Popular scientific summary

- The effect of Garcinia cambogia extract on high-fat diet (HFD)-induced obesity, insulin resistance and hepatic lipid accumulation were evaluated.
- The key component of Garcinia Cambogia was identified.

*Garcinia cambogia*, also known as Malabar tamarind, is native to southeastern Asia (1). It is one of the most popular weight management supplements due to its beneficial effects on weight and fat reduction. Therefore, it is used in natural products for treating obesity and weight control (2, 3). Hydroxycitric acid (HCA) is a major active component of the rind of the fruit *G. cambogia* (4, 5). *G. cambogia* peel supplements contain 20–60% HCA, which may be the reason for their weight loss properties (6–8). HCA is an α-, β-dihydroxytricarboxylic acid that is a potent competitive inhibitor of adenosine triphosphate (ATP) citrate lyase, which is a key enzyme in the synthesis of fatty acids, cholesterol, and triglycerides (TG) (9, 10). ATP-citrate lyase cleaves citrate into oxaloacetate and acetyl-CoA, so HCA eventually limits the number of acetyl-CoA molecules available for fatty acid synthesis. HCA also decreases the accumulation of lipid droplets and the TG level by downregulating fatty acid synthase protein and increasing the phosphorylation of acetyl-CoA carboxylase (11–13). HCA promotes weight loss and inhibits weight gain (without stimulating the central nervous system), stabilizes glucose levels, and improves adipokine levels (14).

Insulin resistance (IR) and non-alcoholic fatty liver disease (NAFLD) caused by high-fat diets (HFDs) are related to metabolic syndrome, including abnormal blood lipid metabolism, elevated TG, elevated low-density lipoprotein, and hepatic lipid accumulation, leading to obesity, type 2 diabetes mellitus (T2DM), and cardiovascular disease (15, 16). Obesity is a continuously growing pandemic that can cause many diseases that affect quality of life (17). According to World Health Organization estimates, > 1.9 billion people aged ≥18 are overweight, and > 650 million of them are obese, which increases the risk of metabolic syndrome (18). Surgical intervention and the use of synthetic drugs for weight loss have limitations and side effects. For example, the anti-obesity drugs orlistat, topiramate, and sibutramine are available on the market, but they have severe side effects such as insomnia, anorexia, dry mouth, and gastrointestinal discomfort, which limit their use (19). Thus, it is key to identify new, effective, and easily tolerated anti-obesity agents from natural products (4, 20–22).

NAFLD is a chronic metabolic stress-related liver injury closely related to genetics and IR; it is one of the clinical consequences of obesity (23). Serious consequences may lead to liver cirrhosis, hepatocellular carcinoma, coronary heart disease and diabetes, and steatosis increases inflammation (24). NAFLD is closely related to lifestyle such as HFD and diseases such as T2DM, dyslipidemia, adrenal hormone excess, obesity, and hypertension (25). The global incidence of NAFLD is increasing. Besides exercise and diet restrictions, there is currently a lack of effective treatment options (26, 27). *G. cambogia* had many activities related to metabolic syndrome because it was able to efficiently improve body fat mass, blood sugar level, body weight, TC, triglyceride level, glucose metabolism and display leptin-like activity (1, 28, 29), and its protective potential against NAFLD has been investigated (30). Despite these findings, to our knowledge the inhibitory effect of ethanol extract of *G. cambogia* in IR and NAFLD remain poorly understood. Therefore, this study explored the effect of GE, GED, and GEP on IR and hepatic lipid accumulation in HFD-induced obese mice.

## Materials and methods

### G. cambogia water extract

*G. cambogia* was purchased from Sri Lanka. The *G. cambogia* peel was subjected to extraction three times by placing it in boiling distilled water for 30 min each time. The solution was collected, concentrated, and lyophilized to obtain GE.

To obtain further extracts, GE and ethanol (1:3) were mixed together and then left to stand. Next, the supernatant was centrifuged. The resulting supernatant was designated the GED. After being left to stand, the precipitate was designated the GEP.

To assess the polysaccharides, total phenols, and flavonoids in GE, we used the phenol–sulfuric acid method, Folin–Ciocalteu method, and NaNO_2_-Al (NO_3_)_3_-NaOH colorimetric method, respectively. High-performance liquid chromatography (HPLC; Agilent 1200 HPLC, Jiangsu Jingmei Biotechnology Co., Ltd.) was used to assess the HCA levels in GE, GED, and GEP.

### Animals and diets

Two animal experiments were conducted. In the first, HFD mice were treated with increasing concentrations of GE (0.2, 1, or 5 g/L GE) and compared to HFD-only control group. In the second experiment, HFD mice given GE, GED, or GEP were compared to HFD-only control group.

Male C57BL/6 mice aged 6–8 weeks were purchased from the Kunming Institute of Botany, Chinese Academy of Sciences and raised at Yunnan Agricultural University. During the entire experiment, the mice were kept in a specific-pathogen-free facility under a 12/12-h light/dark cycle and had free access to food and water (31). The mice were randomly divided into eight groups (six per group), and all were fed HFD (D12492, rodent diet with 60% kcal% fat, Research Diets, Inc.). The first four groups were as follows: HFD only, HFD+GE 0.2 g/L, HFD+GE 1 g/L, and HFD+GE 5 g /L. The second four groups were as follows: HFD only, HFD+GED 4 g/L, HFD+GEP 1 g/L, and HFD+GE 5 g/L. The corresponding extracts were added to the drinking water for 16 weeks. We recorded the weight and fasting blood glucose (FBG) of each mouse every week, as well as the changes in the amount of water and food intake of each mouse. After 16 weeks, the mice were sacrificed, and the liver, serum, inguinal fat, and epididymal fat were collected. All experimental procedures were performed in accordance with the guidelines specified by the Committee for Care and Use of Laboratory Animals of Yunnan Agricultural University and were approved by the Animal Experiments Ethics Committee of Yunnan Agricultural University (SCXY (Chuan) K2020-030) (32).

### Testing tolerances for intraperitoneally injected glucose and insulin

In the 14th week, an intraperitoneal glucose tolerance test (IPGTT) was performed. The mice were fasted for 13 h, and the body weight of each mouse was measured and recorded. Each mouse was intraperitoneally injected with glucose solution (1 g/kg), and the blood glucose was measured at 0, 30, 60, 90, and 120 min with a blood glucose meter. The data were plotted, and the areas under the curve (AUCs) were calculated (33).

In the 15th week, an intraperitoneal insulin tolerance test (IPITT) was performed. The mice were not fasted, and the body weight of each mouse was measured and recorded. Insulin solution (0.75 U/kg) was injected into the intraperitoneal cavity of each mouse, and the blood glucose was measured at 0, 30, 60, 90, and 120 min using a blood glucose meter. The data were plotted, and the AUCs were calculated (34).

### Cholesterol and aminotransferases

An Advia Chemistry XPT automatic biochemical analyzer (Siemens, Munich, Germany) at Kunming First People’s Hospital and Ganmei International Hospital was used to determine serum TC, high-density lipoprotein cholesterol (HDL-C), non-high-density lipoprotein cholesterol (non-HDL-C), aspartate aminotransferase (AST), and alanine aminotransferase (ALT) levels.

### Liver histological analysis

Mouse liver tissue was fixed with 4% paraformaldehyde, embedded in paraffin, subjected to hematoxylin and eosin (HE) staining, and fixed with neutral resin glue. The liver TG was measured using a TG kit (Nanjing Jiancheng Bioengineering Institute, Nanjing, China).

### Statistical analysis

All data in this study are derived from three or more independent experiments. The data are expressed as the means ± standard deviation (SD). GraphPad Prism 9.0 software (GraphPad software, San Diego, CA, USA) was used to determine the statistical significance of differences between groups. The differences were assessed by one-way analysis of variance (ANOVA) and Tukey’s test. *P* < 0.05 was considered statistically significant.

## Results

### GE inhibits HFD-induced body weight gain and IR

**Fig. 1:**
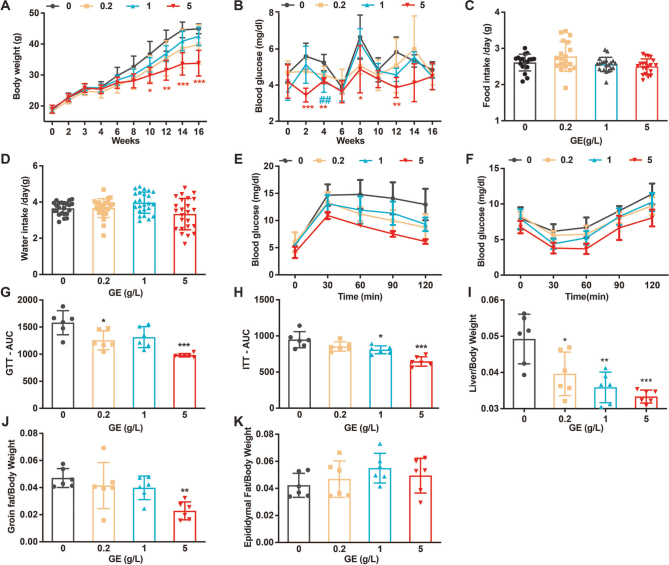
GE reduces HFD-induced obesity and IR. (A) Weight changes of the four groups of HFD mice administered 0, 0.2, 1, or 5 g /L GE for 16 weeks. (B) Weekly fasting blood glucose. (C) Food intake. (D) Water intake. (E) IPGTT results. (F) IPITT results. (G) AUC values for IPGTT. (H) AUC values for IPITT. (I) Liver-to-body weight ratio. (J). Inguinal adipose tissue-to-body weight ratio. (K). Epididymal fat tissue-to-body weight ratio. Data are expressed as mean ± SD. One-way ANOVA and Tukey’s tests were used to analyze statistical differences. *P < 0.05, **P < 0.01, ***P < 0.001, ^##^*P* < 0.01. *HFD vs. HFD+5 g/L, ^#^HFD vs. HFD+1 g/L.

C57BL/6 mice fed HFD were given 0, 0.2, 1, or 5 g /L *G. cambogia* extract (GE) in drinking water for 16 weeks. The mice fed HFD for 16 weeks gained body weight (Fig. 1A) and developed IR (Fig. 1E–H). GE effectively inhibited HFD-induced obesity, and the body weight decrease of the highest-dose group (5 g/L GE) compared to the HFD mice was significant from the 10th week (18.64% lower, *P* < 0.05; 24.67% reductions in the16th, *P* < 0.001, Fig. 1A). GE did not affect the food or water consumption of mice, indicating that the anti-obesity effect of GE was not caused by appetite suppression or a change in water consumption (Fig. 1C–D).

Obesity is often accompanied by abnormal glucose metabolism. Therefore, we further assessed the effects of GE on glucose homeostasis by testing blood glucose levels using IPGTT and IPITT. The blood glucose levels of the HFD mice were elevated, while the highest-dose GE group exhibited significantly decreased FBG levels compared to the HFD mice (Fig. 1B). HFD mice exhibited a significant impairment in glucose tolerance and an increase in IR, as indicated by IPGTT and IPITT, respectively (Fig. 4E–H). GE increased glucose tolerance (a 37.97% reduction AUC in the 5 g/L GE) and decreased IR (a 31.78% reduction AUC in the 5 g/L GE) in HFD mice. GE significantly reduced the liver-to-body weight and inguinal fat-to-body weight ratios (18.64% lower) compared to those in the HFD group, and the high-dose GE group had a highly significant inhibitory effect on both (32.25% lower, *P* < 0.001; 51.46% lower, *P* < 0.01, respectively, Fig. 1I–J). However, GE had no significant effect on the epididymal fat-to-body weight ratio (Fig. 1K).

### GE improves HFD-induced dyslipidemia and liver damage

**Fig. 2:**
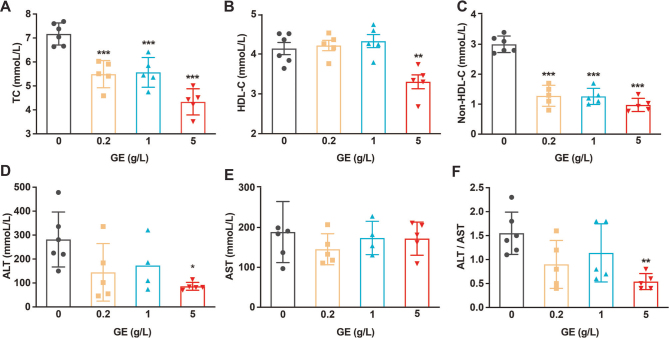
GE reduces dyslipidemia. (A) TC. (B) HDL-C. (C) Non-HDL-C. (D) ALT. (E) AST. (F) ALT/AST. Data are expressed as mean ± SD. One-way ANOVA and Tukey’s tests were used to analyze statistical differences. *P < 0.05, **P < 0.01, ***P < 0.001.

Long-term HFD caused dyslipidemia in mice according to the blood test results. TC and non-HDL-C in the HFD group were abnormally increased by 23.41–39.51%, 57.33–68.40%, and GE significantly improved these factors (Fig. 2A and 2C). Long-term HFD also impaired liver function, which manifested as increased ALT, and 5g/L GE inhibited the HFD-induced increase in ALT (69.40% reduction, *P* < 0.01, Fig. 2D). However, GE did not significantly change the HDL-C or AST levels.

### Effect of GE on HFD-induced hepatic lipid accumulation

**Fig. 3:**
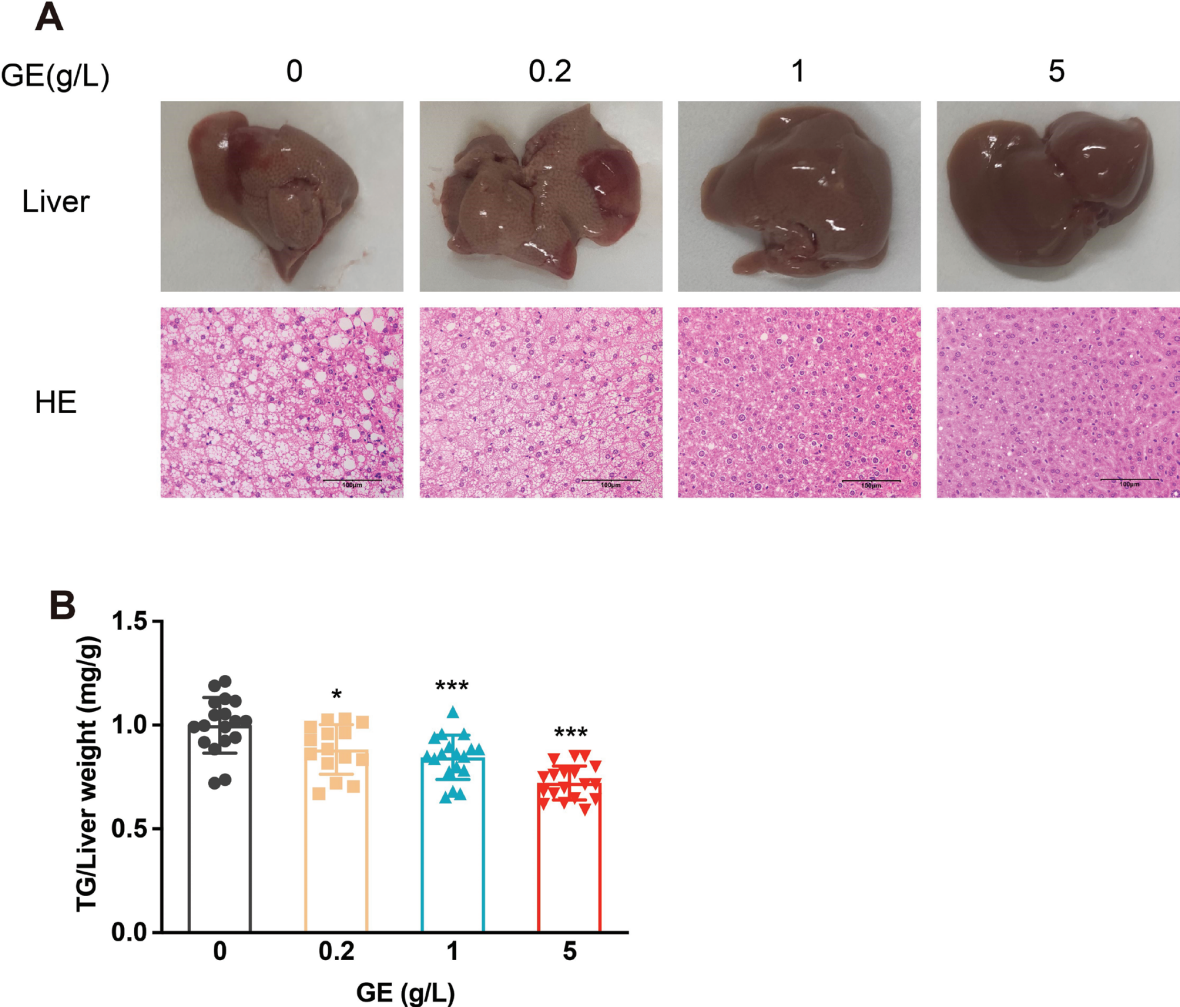
GE improves HFD-induced hepatic lipid accumulation. (A) Liver morphology and HE staining in the four groups of HFD mice administered 0, 0.2, 1, or 5 g /L GE. (B) Hepatic TG level. Data are expressed as mean ± SD. One-way ANOVA and Tukey’s tests were used to analyze statistical differences. *P < 0.05, **P < 0.01, ***P < 0.001.

NAFLD is considered an important public health issue, but there is currently no effective treatment. To assess the effect of GE on HFD-induced hepatic lipid accumulation, mouse liver tissues were subjected to HE staining and TG assessment. GE inhibited the HFD-induced hepatic lipid accumulation (based on the images in Fig. 3A), and dose-dependently significantly inhibited the HFD-induced increase in TG (11.68–27.85%, Fig. 3B).

**Fig. 4:**
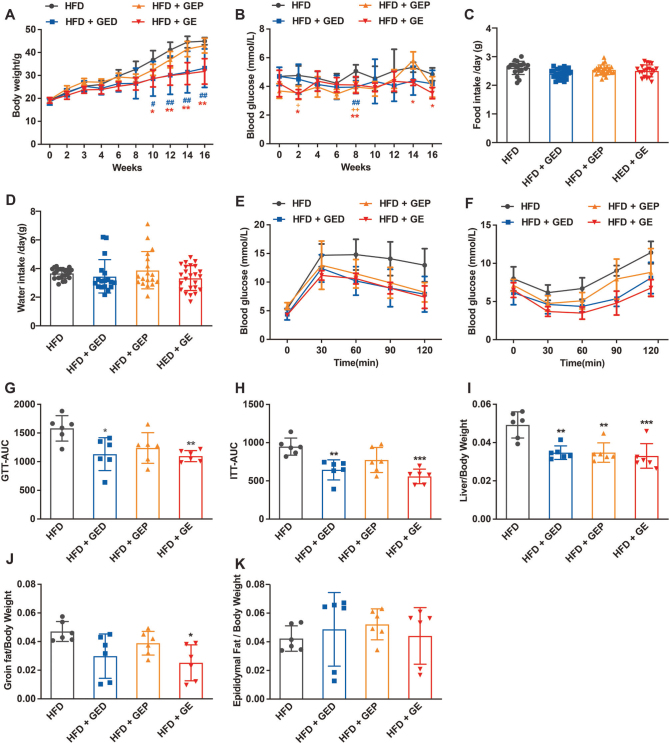
GED and GEP alleviate HFD-induced obesity and IR. GED: 4 g/L, GEP: 1 g/L, GE: 5 g/L. (A) Weight change of the four groups of HFD mice over 16 weeks. (B) Weekly fasting blood glucose. (C) Food intake. (D) Water intake. (E) IPGTT results. (F) IPITT results. (G) AUC values for IPGTT. (H) AUC values for IPITT. (I) Liver-to-body weight ratio. (J) Inguinal adipose tissue-to-body weight ratio. (K) Epididymal fat tissue-to-body weight ratio. Data are expressed as mean ± SD. One-way ANOVA and Tukey’s tests were used to analyze statistical differences. *P < 0.05, **P < 0.01, ***P < 0.001, ^##^*P* < 0.01, ^+^*P* < 0.05, ^++^*P* < 0.01. *HFD versus HFD+GE, ^#^HFD versus HFD+GED, ^+^HFD versus HFD+GEP.

### GED and GEP inhibit HFD-induced obesity and IR

Next, we assessed the effects of the GED or precipitated (GEP) using ethanol. Long-term HFD led to obesity in the mice. Neither GED nor GEP reduced food or water consumption (Fig. 4C–D). The effect of GED regarding body weight reduction was highly significant (22.22–29.39% lower, *P* < 0.01) (Fig. 4A), but that of GEP was not significant. This may be due to the lower dose in the GEP group compared to the GED group (1 g/L vs. 4 g/L), and there was also a slightly lower HCA level in GEP compared to GED (19.79% vs. 22.81%).

To verify the effects of GED and GEP on IR, the mice were subjected to IPGTT and IPITT. The mice in the HFD group developed glucose intolerance and IR. GED and GEP alleviated both of these changes (Fig. 4E–H), and the AUCs for GED (but not GEP) were significantly lower compared to that for the HFD group (GTT and ITT were reduced by 28.49%, 32.07%, *P* < 0.05 and *P* < 0.01, respectively).

GED and GEP significantly inhibited the HFD-induced increase in liver weight by 29.43%, 29.34% (Fig. 4I, *P* < 0.01), and GED reduced inguinal fat (Fig. 4J). GED and GEP had no significant effect on FBG or epididymal fat in mice (Fig. 4B and K).

### GED and GEP alleviate HFD-induced dyslipidemia and liver damage

**Fig. 5:**
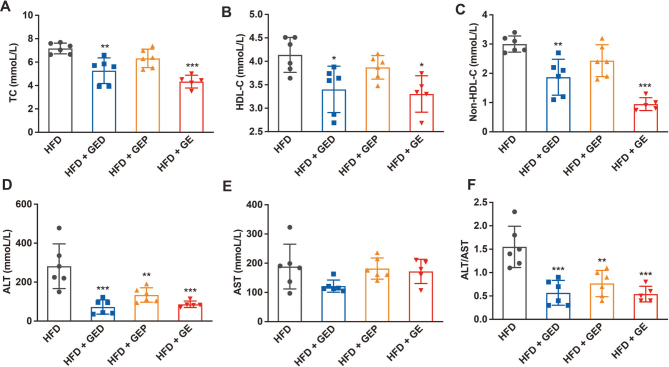
GED and GEP reduce dyslipidemia. GED: 4 g/L, GEP: 1 g/L, GE: 5 g/L. (A) TC. (B) HDL-C. (C) Non-HDL-C. (D) ALT. (E) AST. (F) ALT/AST. Data are expressed as mean ± SD. One-way ANOVA and Tukey’s tests were used to analyze statistical differences, *P < 0.05, **P < 0.01, ***P < 0.001.

The abnormal increases in dyslipidemia and liver damage markers in the HFD group (Fig. 5A–F) are predictors of atherosclerosis and coronary heart disease. TC, HDL-C, non-HCL-C, ALT, and ALT/AST levels were reduced by 25.61, 17.7737.7874.32, and 63.44%, respectively, in the mice fed HFD + GED than in the HFD mice (*P* < 0.05). However, GEP did not significantly inhibit HFD-induced increases in cholesterol (TC, HDL-C, and non-HCL-C) in mice; it only significantly inhibited ALT (52.43% reduction, *P* < 0.01) and ALT/AST (50.54% reduction, *P* < 0.01). This was potentially because the low dose of GEP compared to GED (1 g/L vs. 4 g/L) led to a lower HCA level, along with the slightly lower HCA level in GEP compared to GED (19.79% vs. 22.81%), so the effect was not as obvious.

### GED and GEP inhibit HFD-induced hepatic lipid accumulation

**Fig. 6:**
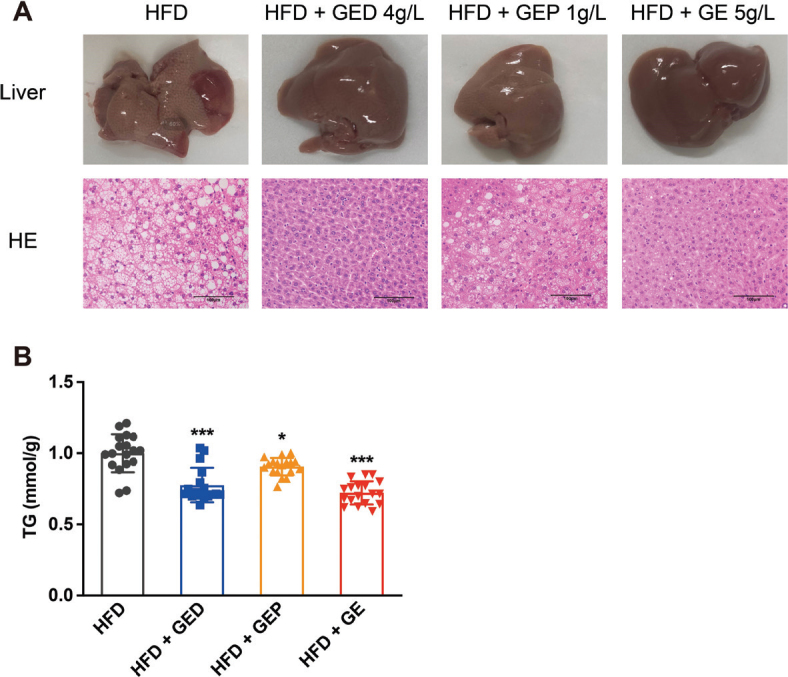
GED and GEP alleviate HFD-induced hepatic lipid accumulation. GED: 4 g/L, GEP: 1 g/L, GE: 5 g/L. (A) Liver morphology and HE staining in the four groups of HFD mice. (B) Liver TG level. Data are expressed as mean ± SD. One-way ANOVA and Tukey’s tests were used to analyze statistical differences. *P < 0.05, **P < 0.01, ***P < 0.001.

HE staining and liver TG assessment showed that GED and GEP alleviated HFD-induced hepatic lipid accumulation (based on the images in Fig. 6A) and significantly inhibited the HFD-induced increase in TG (22.43% reductions in GED, *P* < 0.001; 9.41% reductions in GEP, *P* < 0.05, Fig. 6B). The effect of GED was more significant (*P* < 0.001 vs. *P* < 0.05), which might have been caused by the higher dose in the GED group compared to the GEP group (4 g/L vs. 1 g/L), and there was also a slightly higher HCA level in GED compared to GEP (22.81% vs. 19.79%).

### Levels of polysaccharides, total phenols, flavonoids, and HCA in G. cambogia extract

The contents of polysaccharides, polyphenols, and flavonoids in GE were measured to be 18.88–23.98%, 15.09–17.59%, and 18.39–18.89%, respectively, indicating that the level of polysaccharides in GE was slightly higher. To understand whether the effect of *G. cambogia* on weight, IR, and NAFLD is related to HCA, the HCA levels in GE, GED and GEP were measured by HPLC, and the results were 34.04–35.40%, 22.51–23.11%, and 19.29–20.29%. The level of HCA was the highest in GE, and the level of HCA in GED group was higher than that in GEP group.

## Discussion

NAFLD is one of the most common chronic liver diseases. Thus, exploring how to treat NAFLD using dietary supplements is warranted. We found that (GE, GED, and GEP) alleviated HFD-induced IR and hepatic lipid accumulation in mice. Additionally, it inhibited the HFD-induced increased body weight and inguinal fat in mice, and it also affected the HFD-induced changes in blood test results in mice, especially the changed in TC and non-HDL-C. The HCA levels in GE, GED, and GEP were determined by HPLC, and we speculate that the effects of GE were caused by HCA.

Obesity is a global public health problem, and it is often associated with metabolic diseases such as IR, T2DM, NAFLD, coronary heart disease, and dyslipidemia (35–37). Obesity, caused by fat accumulation, is a serious risk factor for so-called lifestyle-related diseases (38). *G. cambogia*/HCA products are widely marketed as dietary supplements, and *G. cambogia* has been shown to have positive effects regarding reducing body weight, lowering glucose levels, and inhibiting fat synthesis (39–42). Most related studies have shown that *G. cambogia*/HCA can reduce weight gain (9, 43–45) and visceral fat accumulation (46, 47). However, some studies have shown that HCA has no lasting beneficial effect on weight loss, which may be due to the short duration of the study (48). Our research showed that GE inhibited HFD-induced weight gain in mice, but it was not related to food intake (Figs. 1A, 1C, 4A, and 4C), which was different from previous studies showing that *G. cambogia* reduces body weight by suppressing appetite (7, 49). It also inhibited HFD-induced inguinal fat accumulation in mice, but it had no obvious effect on epididymal fat accumulation. Abnormal blood lipid metabolism plays a major role in the whole process of atherosclerosis occurrence, development, and deterioration, and is a major risk factor for coronary heart disease (41). We found that *G. cambogia* significantly reduced the HFD-induced mice serum increases in TC and non-HDL-C. In addition, we found that *G. cambogia* had a better effect on HFD-induced mice serum ALT than AST (Figs. 2D, 2E, 5D, and 5E), indicating that *G. cambogia* may inhibit HFD-induced hepatocyte membrane damage. These findings have potential significance for the treatment of metabolic disorders.

In addition to inducing weight loss, *G. cambogia* relieved IR and hepatic lipid accumulation. IR in metabolic diseases has received considerable attention in recent years (50). It is one of the key components of metabolic syndrome (51, 52). We found that *G. cambogia* alleviates HFD-induced glucose intolerance and IR (Figs. 1E–H and 4E–H), which is consistent with the results of previous studies (29, 53). It was recently reported that *G. cambogia* and HCA activated the antioxidant effect of Nrf2 antioxidant response element and improved NAFLD by regulating adipogenesis and apoptosis (54). Relatedly, using a mouse model of HFD-induced obesity, we found that *G. cambogia* dose-dependently improved hepatic lipid accumulation (Figs. 3 and 6), providing a new option for the treatment of NAFLD.

In conclusion, this study demonstrated that *G. cambogia* alleviates IR and hepatic lipid accumulation and has a weight-loss effect in HFD mice. These effects may be caused by the HCA in *G. cambogia*. Although we did not further assess whether HCA mediates the effects, based on our results, *G. cambogia* still has potential for the treatment of NAFLD.

## Conflict of interest and funding

The authors declare that they have no known competing financial interests or personal relationships that could have appeared to influence the work reported in this paper. This work was supported by the Yunnan Provincial Joint Project of Basic Agricultural Research (ref. 2018FG001-036) and Yunnan International Joint Laboratory of Green Health Food (China and Thailand) (ref. 202204BI090012).

## Authors’ contributions

JS and CF received funding. CF conceived and designed the experiment. JD performed the experiments. JD, HZ, XD, XH, BD, WL, and YT contributed to the analysis and interpretation of the data. JD drafted the first edition of the manuscript, and CF edited the manuscript.

## Ethics statement

The animal study was reviewed and approved by the Animal Experiment Ethics Review Committee of Yunnan Agricultural University.

## Data availability statement

The original contributions presented in the study are included in the article; further inquiries can be directed to the corresponding author/s.
